# Substrate-Borne Vibratory Communication during Courtship in *Drosophila melanogaster*

**DOI:** 10.1016/j.cub.2012.09.042

**Published:** 2012-11-20

**Authors:** Caroline C.G. Fabre, Berthold Hedwig, Graham Conduit, Peter A. Lawrence, Stephen F. Goodwin, José Casal

**Affiliations:** 1 Department of Zoology, University of Cambridge, Downing Street, Cambridge CB2 2EJ, UK; 2 King's College, 21 King's Parade, Cambridge CB2 1ST, UK; 3 Department of Physiology, Anatomy and Genetics, University of Oxford, Sherrington Building, Parks Road, Oxford OX1 3PT, UK

## Abstract

Courtship in *Drosophila melanogaster* has become an iconic example of an innate and interactive series of behaviors [1–11]. The female signals her acceptance of copulation by becoming immobile in response to a male's display of stereotyped actions. The male and female communicate via vision, air-borne sounds, and pheromones [1, 2], but what triggers the female's immobility is undetermined. Here, we describe an overlooked and important component of *Drosophila* courtship. Video recordings and laser vibrometry show that the male abdomen shakes (“quivers”), generating substrate-borne vibrations at about six pulses per second. We present evidence that the female becomes receptive and stops walking because she senses these vibrations, rather than as a response to air-borne songs produced by the male fluttering the wings [1, 2, 12]. We also present evidence that the neural circuits expressing the sex-determination genes *fruitless* and *doublesex* [8] drive quivering behavior. These abdominal quivers and associated vibrations, as well as their effect on female receptivity, are conserved in other *Drosophila* species. Substrate-borne vibrations are an ancient form of communication that is widespread in animals. Our findings in *Drosophila* open a door to study the neuromuscular circuitry responsible for these signals and the sensory systems needed for their reception.

## Highlights

► Courting *Drosophila* males vibrate their abdomen generating substrate-borne signals ► Females respond to these signals by stopping movement, to allow copulation ► This substrate-borne mode of communication is conserved among *Drosophila* species ► *fruitless* and *doublesex* expressing neurons underlie the abdominal vibration behavior

## Results and Discussion

### Characteristics of Male Quivering during Courtship

Pairs of flies were placed in a chamber and filmed at 30–150 frames per second. The behaviors of both the male and female were annotated and analyzed from initiation of courtship until copulation. In addition to well-known courtship behaviors, we observed frequent bouts of abdominal movements in the male that we refer to as “quivering” (Movie S1 available online). Quivering consists of up-and-down movements of the abdomen (Movie S2) with a frequency of 6.64 ± 0.78 beats per second (n = 12 bouts/12 flies). Quivering is a behavior specific to male courtship: we find that females and isolated males do not quiver, nor do males placed with other males (data not shown).

**Figure 1:**
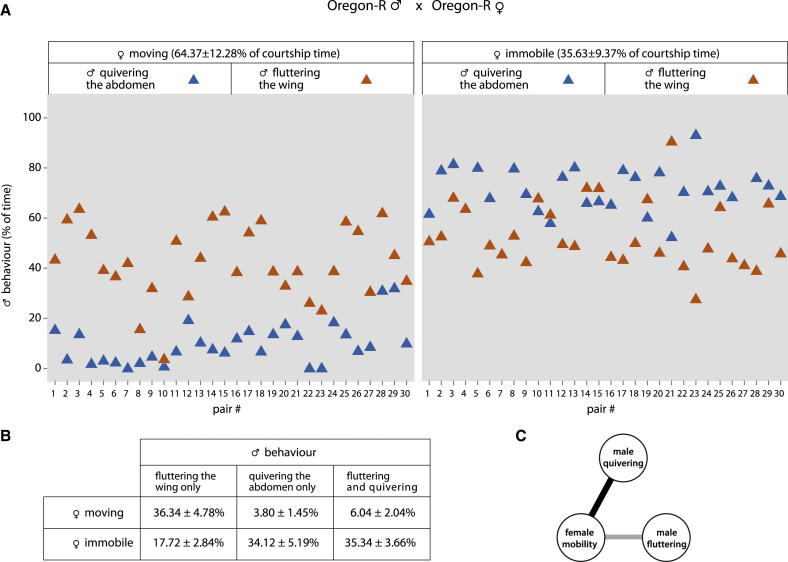
Two Behaviors of the Male Vary in Frequency with Respect to whether the Female Is Moving or Immobile (A and B) Frequencies were extracted from the ethograms built from movies of courting pairs. In (A), the x axis shows the values for each of 30 pairs of Oregon-R flies. The y axis shows the percentage of the time the males display wing fluttering (including wing extension/vibration and scissoring) or abdominal quivering. The left scatterplot shows these two male behaviors when the females are moving, and the right one the male behaviors when the females are stationary. Note that one male behavior is shown without indicating whether the same male is also performing the other behavior at that time. Therefore, the table in (B) breaks down male behavior further, showing for each behavior the grand means (n = 30, as before) as percentage of the time the female is moving or immobile. See also Figures S1A and S1B. All intervals in this report are given for a 95% confidence level. (C) Log-linear models of association were tested (see Figure S1D), and the best fit includes a strong association between male quivering (or not) and female movement (or not) and a weaker but still significant association between male fluttering (or not) and female movement (or not). See also Figures S1 and S2, Movie S1, and Movie S2.

We quantified 30 ethograms of completed courtships (Experimental Procedures) in wild-type Oregon-R flies (Figures 1 and S1) for courtship behaviors including wing fluttering alone (about one-third of total courtship time), abdominal quivering alone (one-seventh of total courtship time), and simultaneous wing fluttering and abdominal quivering (one-seventh of total courtship time). We also recorded whether females were moving or stationary (Figure 1); they were immobile for one-third of the total courtship time (Figure S1B).

The most interesting results come from when the two behaviors of the males are compared relative to the simultaneous behavior of the female (Figure 1). Male quivering occurs 69% of the time that females are stationary but only 10% of the time that females are moving (Figure 1). In contrast, males flutter their wings about as often, independently of whether females are stationary or moving (53% and 42%, respectively; Figure 1). Although males flutter their wings for approximately half of the time that the females are stationary (53%), we note that males are also quivering for two-thirds of this 53%. However, when males flutter during female movement (42%), the males quiver for only one-seventh of this 42% (Figure 1). Thus, the data show that female immobility can coincide with male fluttering but mainly occurs when he is also quivering. Statistical analysis of the data shows that male quivering behavior (quivering or not) and female behavior (stationary or moving) are strongly associated, whereas the comparable association between male fluttering and female behavior also exists but is weaker (Figure 1 and S1C). Similar results were found for male and female pairs of another wild-type strain, Canton S (Figures S1E–S1G).

Our results do not support the general perception that signals generated by male wing fluttering act alone to diminish movement of females [7]. They show instead that quivering of the abdomen coincided with female immobility much more than wing fluttering did (Figure 1 and Movie S1). Also, and consistent with this finding, bouts of quivering vary in duration and depend on whether the female is moving (average duration only 1 s; Figure S1B) or stationary (average duration about 3 s; Figure S1B). In contrast, bouts of fluttering were longer when the female was moving than when she was immobile (Figure S1B).

If the wings of the wild-type male were amputated, males quivered more frequently than the wild-type (t test, p = 0.011) and for longer periods (p = 0.029); females placed with these males stopped moving more than when paired with intact males (p = 0.017) (Figures S2A–S2C). We also used males carrying mutations in the sex-determination genes *doublesex* (*dsx*) and *fruitless* (*fru*) because neurons expressing these genes drive male-specific behavior [5, 13, 14]. These mutations had no clear effects on the percentage of the courtship time that males fluttered their wings (*dsx*^*–*^ p = 0.45 and *fru*^*–*^ p = 0.24), although the pattern of fluttering was different from the wild-type (data not shown). However, *dsx*^*–*^ (Figures S2D–S2F) and *fru*^*–*^ (Figures S2G–S2I) mutant males quivered less than normal males (*dsx*^*–*^ p = 9.27 × 10^−5^ and *fru*^*–*^ p = 2.17 × 10^−9^). Importantly, we observed that their wild-type female partners stopped less than when courted by wild-type males (*dsx*^*–*^ p *=* 0.026 and *fru*^*–*^ p = 0.006) (Figures S2D–S2I, compare with Figure 1). Thus, if the male quivering is increased or decreased by intervention, the wild-type females stop more or less often, respectively (Figures S2A–S2I). These findings argue (but do not prove) that female stopping is a response to quivering and not a cause of quivering.

A mild activation of *dsx*-expressing neurons or *fru*-expressing neurons by forcing expression of *Drosophila* TRPA1 (Movie S3) triggered quivering in solitary males—as well as a mélange of other courtship behaviors (Movie S3) [15]. When stronger conditions were used to activate the *fru*-expressing neurons, quivering was induced also in females, arguing that appropriate neurons and circuitry are present but latent in the female. It follows that some of these neurons direct the abdominal quivering of the male during courtship and that the neuronal circuitry differs between normal males and females.

### How Might Females Sense Male Quivering?

Our observations suggest that quivering of the male abdomen is sensed by the female and causes her to stop walking. We therefore asked how the tremor of the male abdomen might be transmitted to the female. One possibility is that the female could see quivering—vision is known to be used during courtship [7, 9]. To investigate, we performed courtship assays in the “dark” using infrared light that flies cannot detect [16]. Males quivered normally and again there was a strong coincidence between quivering and female immobility, suggesting that vision is not an important component (Figures S2J–S2L).

**Figure 2:**
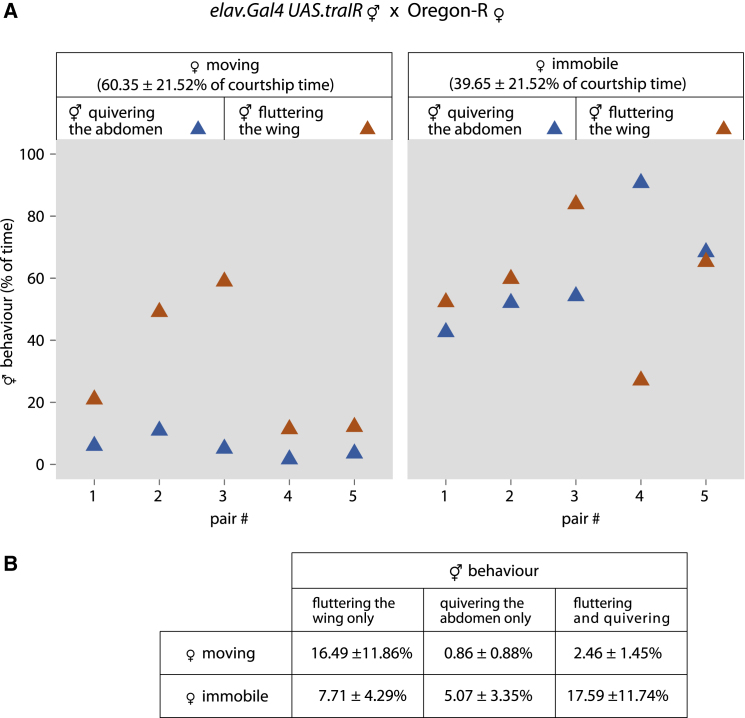
Behaviors of Neuronally Masculinized Females during Courtship with Wild-Type Females Masculinized females, like wild-type males, quiver their abdomens and the wild-type female partners appear to respond by stopping. Data are presented as in Figure 1. See also Figure S3 and Movie S4.

Next, we asked whether quivering might be associated with release of male-specific pheromones via the cuticle. In *Drosophila*, pheromones are low-volatility hydrocarbons and are produced by the abdominal oenocytes of both male and female [7]. By using RNA interference, we reduced the expression of the sex-determination gene *transformer* (*tra*), but only in the female nervous system [17]. The result was neuronally masculinized females that showed male-like behavior directed toward normal females; these masculinized females exhibited abdominal quivering, which wild-type females never do (Figures 2 and S3 and Movie S4). Their wild-type female partners tend to become immobile when the masculinized females exhibit abdominal quivering (for quantitation, see Figures 2 and S3). We have presented evidence that, in normal courtship and as a response to the male quivering, the females tend to stop. But these neuronally masculinized females have a female anatomy, and accordingly their oenocytes produce only female pheromones [18]. Yet, when they quiver, their wild-type female partners tend to stop moving; we deduce that male pheromones are not the relevant signal emitted during quivering.

**Figure 3:**
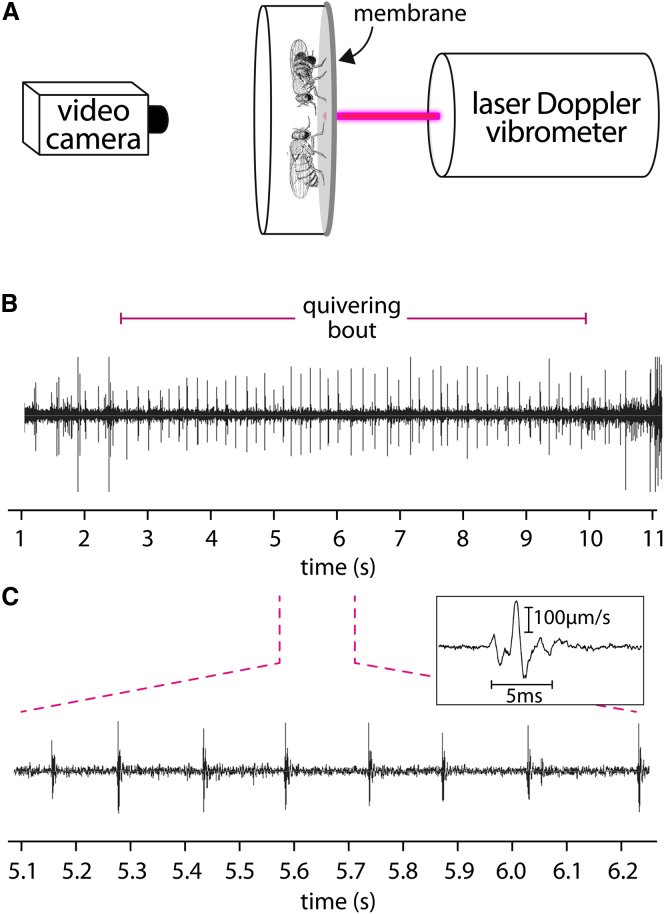
Substrate-Borne Vibrations Generated during Abdominal Quivering of Courting Males (A) Scheme of the video and laser vibrometer recording system. (B) Oscillogram of substrate-borne vibrations generated during a single bout of quivering of about 7 s; the wings of the male were amputated. There is some variation in the amplitudes of the substrate vibrations. (C) Details of (B) above to show higher resolution. See also Movie S5.

Could abdominal quivering generate an acoustical signal? It seems that it does not; a variety of detectors, including insectavox microphones [19] (Experimental Procedures), which we used successfully to record the wing song, all failed to detect any air-borne sound associated with quivering (data not shown). To determine whether quivering generates substrate-borne vibrations, the courting pair was placed on a membrane; so that any possible vibrations caused by wing fluttering could be avoided, the male's wings were amputated. A laser vibrometer was used to measure any oscillations of the membrane (Figure 3A). The results show that bouts of quivering coincided precisely with rhythmic vibrations of the substrate (Figures 3B and 3C and Movie S5). Pulse-like vibrations occurred during quivering with a repetition rate of 6.44 ± 0.32 pulses per second (n = 225 pulses/10 flies; Figures 3B and 3C), with each pulse lasting for about 5 ms (Figure 3C). These fit well with the visual analysis (6.64 ± 0.78 beats per second), arguing that each beat corresponds with a single pulse. There is considerable regularity in the time intervals between pulses (Figure 3), giving a pulse interval of 165.61 ± 4.21 ms (n = 225 pulses/10 flies). There is some indication of variation in the amplitude of the abdominal beats during quivering (Movie S2), which may correspond with the observed variation in amplitude of the substrate vibration pulses. In some arthropods that employ substrate-borne vibratory communication during courtship, patterned variation in amplitude may assist species recognition [20], raising the possibility that *Drosophila* does the same. In *Drosophila*, as in other arthropods, tremulations may be transmitted via the legs of the male to the substrate. The substrate-borne vibrations that result may be perceived by the female either by chordotonal organs present on the proximal tibia or carried through the body to the Johnston's organ at the base of the antenna [20–22], or in both of these ways.

During courtship several behaviors may act synergistically to aid copulation. Our evidence argues that quivering is particularly important because it correlates strongly with the female ceasing movement, considered to be a sign of receptivity [7]. Furthermore, quivering may explain why males of, for example, *D. heteroneura*, *D. melanogaster*, and *D. silvestris* that have amputated wings or an otherwise impaired serenade can still elicit acceptance by the female [1, 23–25].

**Figure 4:**
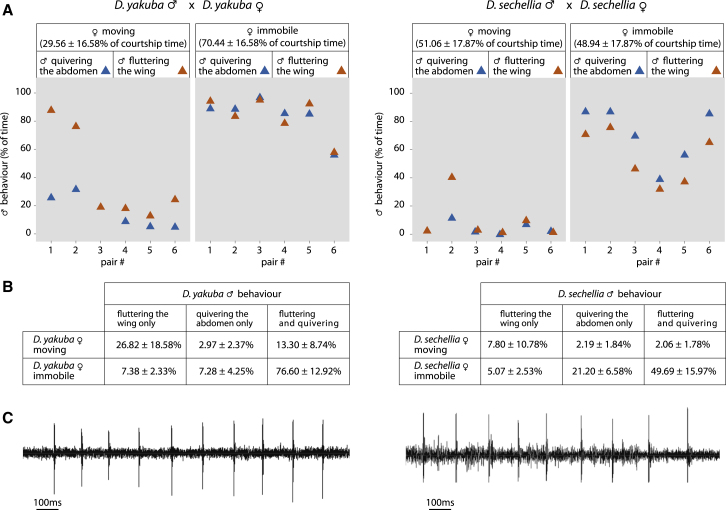
Behaviors of Courting *Drosophila yakuba* and *D. sechellia* Pairs An association between male quivering and female immobility is apparent. (A and B) Data are presented as in Figure 1. (C) Oscillograms of substrate-borne vibrations generated during a single bout of quivering show that the vibrations are similar to *D. melanogaster*. See also Figure S4 and Movie S6.

We found both abdominal quivering and associated substrate-borne vibrations are conserved in other *Drosophila* species (Figure 4). In *D. sechellia* and *D. yakuba* (Figures 4A, 4B, S4A, and S4B, and Movie S6), we observed vibrations in the substrate with a pulse repetition rate of 7.13 ± 0.96 (*D. sechellia*; n = 50 pulses/5 flies) and 6.80 ± 0.49 (*D. yakuba*; n = 19 pulses/5 flies) (Figure 4C). The pulse interval is 157.56 ± 11.13 ms (n = 50 pulses/5 flies) for *D. yakuba* and 173.37 ± 8.70 ms (n = 19 pulses/5 flies) for *D. sechellia.* The frequency and length of the quivering bouts varied (data not shown). In both species, males simultaneously quiver the abdomen and flutter their wings more frequently than *D. melanogaster* (Figures 4, S4A, and S4B; compare with Figures 1 and S1). Importantly, in both species, quivering was again strongly associated with female immobility (Figure 4 and Movie S6). Less-detailed, yet similar, observations were made on different *Drosophila* species: some from the same group as *D. melanogaster* (*D. biarmipes*, *D. mauritiana*, and *D. simulans*), and others from more distant groups (*D. mojavensis* and *D. willistoni*).

It is strange that substrate-borne signals have so far been overlooked in *D. melanogaster*, particularly as substrate-borne vibrations are well known in small invertebrates [26–28]. Tremulatory signals were detected during courtship of other arthropods, for example pentatomid bugs [20] and salticid spiders [22, 29, 30]. Such signals may be generated by up-and-down movements of the abdomen, similar to the quivering we observe, or by shaking of appendages [28, 29, 31–35]. Substrate-borne vibrations were thought to be unusual in Diptera; exceptions were the male and female reed fly, *Lipara*, which exchange signals as vibrations transmitted within the reed stems. The male signal appears to originate from tremulations of the abdomen [36]. Even two decades ago, abdominal movements were observed in *D. silvestris*, but this was then reported as a behavior unique to these flies of the Hawaiian islands [37]. Later, a repeated movement of the male abdomen that tapped the substrate was noted as part of a broad description of courtship behavior in *D. melanogaster* but not associated with any particular female behavior [38]. We have now characterized a male behavior, which we call quivering, that does not appear to include contact with the substrate and that generates substrate-borne vibrations. We do not know exactly how quivering produces these vibrations, but notice that the pulses themselves are short, suggesting some instantaneous element within the quiver beat.

The characteristics of substrate-borne signals depend on the material in which they are transmitted; they are robust and can propagate with little attenuation [21]. The frequency, amplitude, and modulation of these vibrations may carry information to the receiver about the sender [27, 39–41]. Substrate-borne signals may not be detectable by predators, as the latter may not possess suitable receptors [42]. It has not escaped our notice that vertebrates also use substrate-borne vibratory signaling [43].

## Experimental Procedures

### Fly Mutant and Wild-Type Stocks

Flies were raised on standard cornmeal medium under a 12:12 hr light:dark cycle and kept and tested at 25°C with 65% humidity. For the analysis of wild-type behavior, we used Oregon R (OrR) and Canton S (Cs). *fru.Gal4* (*fru*^*Gal4.P1.D*^) and *elav.Gal4* (*elav*^*c155*^) flies were obtained from the Bloomington Stock Center. *UAS.dTRPA1* flies were kindly provided by Stefan Pulver. *UAS.traIR* flies were obtained from the VDRC Stock Center. The *dsx.Gal4* (*dsx*^*Gal4.KI*^) line used was that described in [44]. For the analysis of the effect of mutations in the sex-determination genes, two allelic combinations were used: *dsx*^*1*^*/Df(3R)dsx15* and *fru*^*ΔC*^*/Df(3R)4-40*. For details of mutant alleles, see FlyBase [45]. *Drosophila simulans*, *D. yakuba*, *D. mauritiana*, *D. sechellia*, *D. biarmipes*, and *D. willistoni* flies were obtained from the University of California *Drosophila* Species Stock Center. *D. mojavensis* flies were kindly provided by Darren Parker. Adult flies were collected upon eclosion with light CO_2_ anesthesia. Before mating, individual males or small groups of five to ten virgin females were kept isolated in vials with fresh food. For some experiments, courting pairs were kept under infrared light [16] or the wings of collected males were cut off with microscissors and under anesthesia.

### Behavioral Recording

Pairs of flies were tested in a single trial when they were 4–6 days old. Their behavior was recorded with a 10× macro lens and a Firewire Stingray F-033B camera (Allied Vision Technologies; Stadtroda, Germany) and acquired with “Astro IIDC” (Aupperle Services and Contracting; Calgary, Canada) into a laptop computer. For analysis of the wild-type, 30 courting pairs were recorded and analyzed. For other studies, a minimum of four pairs of flies was tested. Transparent plexiglass courtship chambers (10 mm diameter and 6 mm height) were assembled from two half chambers each of 3 mm height. Each fly was collected with a mouth aspirator and introduced into one half chamber. After a recovery period of 5 min, both halves were fused, and filming of the pair was commenced. Recording was started at the initiation of courtship and for approximately 600 s, or until copulation occurred. Each pair was tested only once. Before each test, chambers were washed with ethanol and dried.

### Heat-Activation Experiments

Ectopic expression of the heat-activatable cation channel TRPA1 (dTRPA1) was obtained with the *fru.Gal4* and *dsx.Gal4* drivers in both males and females. The courtship chamber was inserted into a metal heating block set to produce a temperature of 26°C–27°C inside the chamber; at this temperature, we observed an effect on male but not female behavior with both drivers (Movie S3). We noted that at 29.5°C and only using the *fru.Gal4* driver, the females began to quiver; however, the male's behavior became even more frenetic [15].

### Behavior Annotations and Analysis

Movies were annotated with the “Annotation” software version 1.3, registering all standard male courting behaviors (such as orientating toward the female, following the female, proboscis extension, licking, tapping), in particular when males showed wing fluttering (this behavior comprises wing extension/vibration and scissoring) and/or abdominal quivering, and also whether the female was moving or immobile. The data for each movie were imported into Excel files. For statistical analysis and generation of diagrams, we used the R programming language and software environment [46]. All intervals shown in the paper are for 95% confidence level. We tested for associations between the three behavioral variables: female mobility, male quivering, and male fluttering; using the number of bouts, *N*, we fitted a series of generalized linear models with a Poisson error structure, *N ∼female mobility +* (or) _*^∗^*_ *male quivering +* (or) _^∗^_ *male fluttering* [47] (see legends to Figures S1C and S1D).

### Recording Vibrational Signals with Laser Vibrometry

Video and laser vibrometer recordings were conducted on a vibration-damped table in a soundproof room. Flies were placed into cylindrical chambers of approximately 10 mm in diameter and 6 mm in height, made of plastic. The top of this cylinder was a transparent film through which the flies were recorded using the Stingray F-33B camera with an attached blue filter (cutoff wavelengths at 395 and 480 nm). The bottom of the cylinder consisted of a piece of thermal foil, a membrane made of silver metallized polyester material, with an albedo of approximately 0.8 (Sub Zero Technology; Leicester, UK). The beam of a PSV-400 laser vibrometer (Polytec. Waldbronn, Germany) was directed perpendicular to the surface of this membrane (Figure 3). Signals were digitized with 12 bit amplitude resolution with a PCI MIO-16-E4 card (Analog Devices; Norwood, MA) and digitized with LabView (National Instruments; Austin, TX) on a PC. Signals were transformed into .wav data with the Neurolab software [48]. Video and laser vibrometer recordings were synchronized at the start by brief interruption of the laser path; this produces both a momentary peak in the oscillogram and a black frame in the video. Oscillograms were analyzed with the Raven software [49]. Neither an electret microphone (frequency response, 50 Hz to 13k Hz; sensitivity, 60 ± 3 dB) nor a piezoelectric transducer (resonant frequency, 2.8 ± 0.5 kHz) registered any air-propagated sound emitted during abdominal quivering. We do not know whether wing fluttering of *Drosophila* produces vibrational signals in the substrate.

## Supplemental Information

Movie S1. A Video Clip of a Pair of Courting *Drosophila melanogaster*, Related to Figure 1 First, the male chases the female and he flutters. Then he quivers, and she stops and cleans her legs. He moves a little closer to her and continues to quiver and flutter. She stops all activity, he quivers and later flutters. Then he moves toward her with abdomen curved and his wings fluttering, and she moves away. Played in real time.

Movie S2. A Video Clip Showing a Male Quivering during Courtship, Related to Figure 1 Quivering consists of vertical abdominal oscillations of low amplitude. The clip covers about 2 s of real time and is played at half speed.

Movie S3. Video Clips of a Solitary *dsx.Gal4, UAS. dTRPA1* Male, Left, and a Solitary *fru.Gal4, UAS.dTRPA1* Male, Right, Related to the Results and Discussion and the Experimental Procedures At the restrictive temperature, both types of males display several courtship behaviors, including quivering, even though they are solitary.

Movie S4. Video Clip of Interaction between a Behaviorally Masculinized *elav.Gal4, UAS.traIR* Female and an Oregon-R Female, Related to Figure 2 The masculinized female (on the left) courts the wild type female and flutters, and she also displays bouts of quivering. The wild-type female is immobile.

Movie S5. Video Clip Showing Simultaneous Quivering and Substrate-Borne Vibrations, Related to Figure 4 The male quivers and generates characteristic vibrations (see also Figure 3). To reduce interference with the oscillogram, the wings of the male have been amputated. The sound you hear is a transformation of the oscillogram record to audible frequencies; it is only there to help you link the oscillogram record with the video.

Movie S6. Video Clips of Abdominal Quivering Displayed by Males in Courting Pairs of *D. yakuba* and *D. sechellia*, Related to the Results and Discussion

## References

[1] Greenspan R.J., Ferveur J.F. (2000). Courtship in *Drosophila*. Annu. Rev. Genet..

[2] Tauber E., Eberl D.F. (2003). Acoustic communication in *Drosophila*. Behav. Processes.

[3] Billeter J.C., Rideout E.J., Dornan A.J., Goodwin S.F. (2006). Control of male sexual behavior in *Drosophila* by the sex determination pathway. Curr. Biol..

[4] Dahanukar A., Ray A. (2011). Courtship, aggression and avoidance: pheromones, receptors and neurons for social behaviors in *Drosophila*. Fly (Austin).

[5] Dauwalder B. (2011). The roles of *fruitless* and *doublesex* in the control of male courtship. Int. Rev. Neurobiol..

[6] Dickson B.J. (2008). Wired for sex: the neurobiology of *Drosophila* mating decisions. Science.

[7] Ferveur J.F. (2010). *Drosophila* female courtship and mating behaviors: sensory signals, genes, neural structures and evolution. Curr. Opin. Neurobiol..

[8] Siwicki K.K., Kravitz E.A. (2009). *Fruitless, doublesex* and the genetics of social behavior in *Drosophila melanogaster*. Curr. Opin. Neurobiol..

[9] Yamamoto D., Jallon J.M., Komatsu A. (1997). Genetic dissection of sexual behavior in *Drosophila melanogaster*. Annu. Rev. Entomol..

[10] Yamamoto D. (2008). Brain sex differences and function of the *fruitless* gene in *Drosophila*. J. Neurogenet..

[11] Kohatsu S., Koganezawa M., Yamamoto D. (2011). Female contact activates male-specific interneurons that trigger stereotypic courtship behavior in *Drosophila*. Neuron.

[12] Tompkins L., Gross A.C., Hall J.C., Gailey D.A., Siegel R.W. (1982). The role of female movement in the sexual behavior of *Drosophila melanogaster*. Behav. Genet..

[13] Villella A., Gailey D.A., Berwald B., Ohshima S., Barnes P.T., Hall J.C. (1997). Extended reproductive roles of the fruitless gene in *Drosophila melanogaster* revealed by behavioral analysis of new *fru* mutants. Genetics.

[14] Villella A., Hall J.C. (2008). Neurogenetics of courtship and mating in *Drosophila*. Adv. Genet..

[15] Pan Y., Robinett C.C., Baker B.S. (2011). Turning males on: activation of male courtship behavior in *Drosophila melanogaster*. PLoS ONE.

[16] Frank K.D., Zimmerman W.F. (1969). Action spectra for phase shifts of a circadian rhythm in *Drosophila*. Science.

[17] Chan Y.B., Kravitz E.A. (2007). Specific subgroups of Fru^M^ neurons control sexually dimorphic patterns of aggression in *Drosophila melanogaster*. Proc. Natl. Acad. Sci. USA.

[18] Fernández M.P., Chan Y.B., Yew J.Y., Billeter J.C., Dreisewerd K., Levine J.D., Kravitz E.A. (2010). Pheromonal and behavioral cues trigger male-to-female aggression in *Drosophila*. PLoS Biol..

[19] Gorczyca M., Hall J.C. (1987). The INSECTVOX, an integrated device for recording and amplifying courtship songs. Drosoph. Inf. Serv..

[20] Cokl A. (2008). Stink bug interaction with host plants during communication. J. Insect Physiol..

[21] Hill P.S. (2009). How do animals use substrate-borne vibrations as an information source?. Naturwissenschaften.

[22] Virant-Doberlet M., Cokl C. (2004). Vibrational communication in insects. Neotrop. Entomol..

[23] Ewing A.W. (1964). The influence of wing area on the courtship behaviour of *Drosophila melanogaster*. Anim. Behav..

[24] Kulkarni S.J., Steinlauf A.F., Hall J.C. (1988). The dissonance mutant of courtship song in *Drosophila melanogaster*: isolation, behavior and cytogenetics. Genetics.

[25] Boake C.R.B., Poulsen T. (1997). Correlates versus predictors of courtship success: courtship song in *Drosophila silvestris* and *D. heteroneura*. Anim. Behav..

[26] Barth F.G., Hoy R.R., Popper A.N., Fay R.R. (1998). The vibrational sense of spiders.

[27] Elias D.O., Mason A.C., O'Connell-Rodwell C.E. (2011). Signaling in variable environments: Substrate-borne signaling mechanisms and communication behavior in spiders. The Use of Vibrations in Communication: Properties, Mechanisms and Function across Taxa.

[28] Hill P.S.M. (2008). Vibrational Communication in Animals.

[29] Elias D.O., Mason A.C., Maddison W.P., Hoy R.R. (2003). Seismic signals in a courting male jumping spider (*Araneae: Salticidae*). J. Exp. Biol..

[30] Barth F.G. (2002). Spider senses - technical perfection and biology. Zoology (Jena).

[31] Elias D.O., Land B.R., Mason A.C., Hoy R.R. (2006). Measuring and quantifying dynamic visual signals in jumping spiders. J. Comp. Physiol. A Neuroethol. Sens. Neural Behav. Physiol..

[32] Barth F.G. (2001). A Spider's World: Senses and Behavior.

[33] Cocroft R., Rodriguez R. (2010). Host shifts and signal divergence:mating signals covary with host use in a complex of specialized plant-feeding insects. Biol. J. Linn. Soc. Lond..

[34] Rovner J.S. (1980). Vibration in *Heteropoda venatoria* (*Sparassidae*) – a 3rd method of sound production in spiders. J. Arachnol..

[35] Uetz G.W., Stratton G.E., Wittn P.N., Rovner J.S. (1982). Acoustic communication and reproductive isolation in spiders. Spider Communication: Mechanisms and Ecological Significance.

[36] Mook J.H., Bruggemann C.G. (1968). Acoustical communication by *Lipara lucens* (*Diptera, Chloropidae*). Entomol. Exp. Appl..

[37] Hoy R.R., Hoikkala A., Kaneshiro K. (1988). Hawaiian courtship songs: evolutionary innovation in communication signals of *Drosophila*. Science.

[38] Lasbleiz C., Ferveur J.F., Everaerts C. (2006). Courtship behaviour of *Drosophila melanogaster* revisited. Anim. Behav..

[39] Dierkes S., Barth F.G. (1995). Mechanism of signal production in the vibratory communication of the wandering spider *Cupiennius getazi* (*Arachnida, Araneae*). J. Comp. Physiol. A Neuroethol. Sens. Neural Behav. Physiol..

[40] Markl H., Huber F., Markl H. (1983). Vibrational communication. Neuroethology and Behavioral Physiology.

[41] Michelsen A., Fink F., Gogala M., Traue D. (1982). Plants as transmission channels for insect vibrational songs. Behav. Ecol. Sociobiol..

[42] McGregor P.K. (2005). Animal Communication Networks.

[43] Caldwell M.S., Johnston G.R., McDaniel J.G., Warkentin K.M. (2010). Vibrational signaling in the agonistic interactions of red-eyed treefrogs. Curr. Biol..

[44] Rideout E.J., Dornan A.J., Neville M.C., Eadie S., Goodwin S.F. (2010). Control of sexual differentiation and behavior by the *doublesex* gene in *Drosophila melanogaster*. Nat. Neurosci..

[45] McQuilton P., St Pierre S.E., Thurmond J., FlyBase Consortium (2012). FlyBase 101—the basics of navigating FlyBase. Nucleic Acids Res..

[46] R Development Core Team (2011). R: A Language and Environment for Statistical Computing.

[47] Friendly M. (1999). Extending mosaic displays: marginal, conditional, and partial views of categorical data. J. Comput. Graph. Statist..

[48] Hedwig B., Knepper M. (1992). NEUROLAB, a comprehensive program for the analysis of neurophysiological and behavioural data. J. Neurosci. Methods.

[49] Bioacoustics Research Program (2004). Raven Pro: Interactive Sound Analysis Software. Version 1.2 Edition.

