# Supplementary figures and images for: Substrate-Borne Vibratory Communication during Courtship in Drosophila melanogaster
Source: Curr Biol. 2012 Nov 20;22(22):2180–5. doi: 10.1016/j.cub.2012.09.042 (PMC3502867; doi:10.1016/j.cub.2012.09.042)

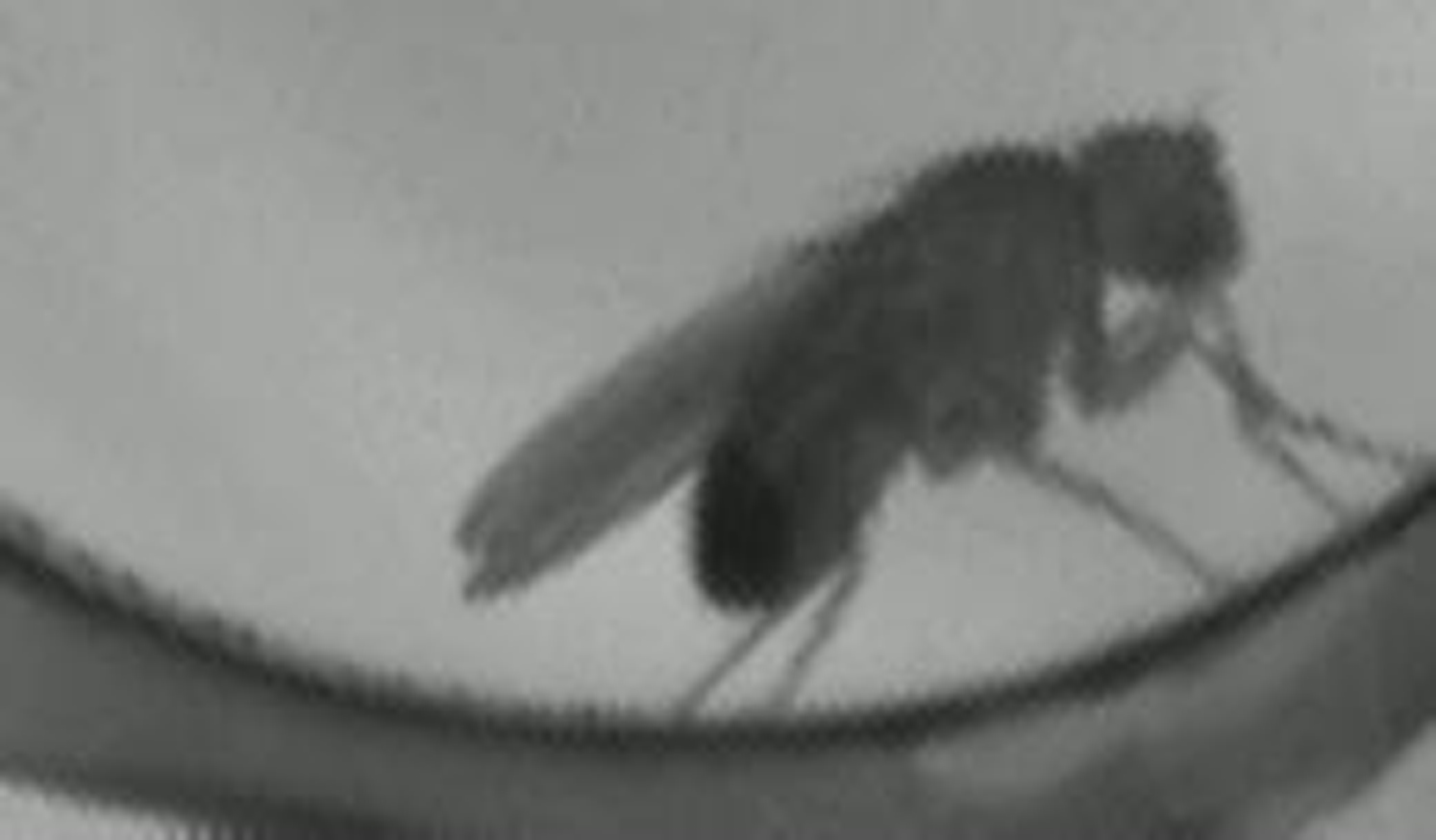

Supplement: Movie S2. A Video Clip Showing a Male Quivering during Courtship, Related to Figure 1 — Quivering consists of vertical abdominal oscillations of low amplitude. The clip covers about 2 s of real time and is played at half speed. [file mmc2.jpg]

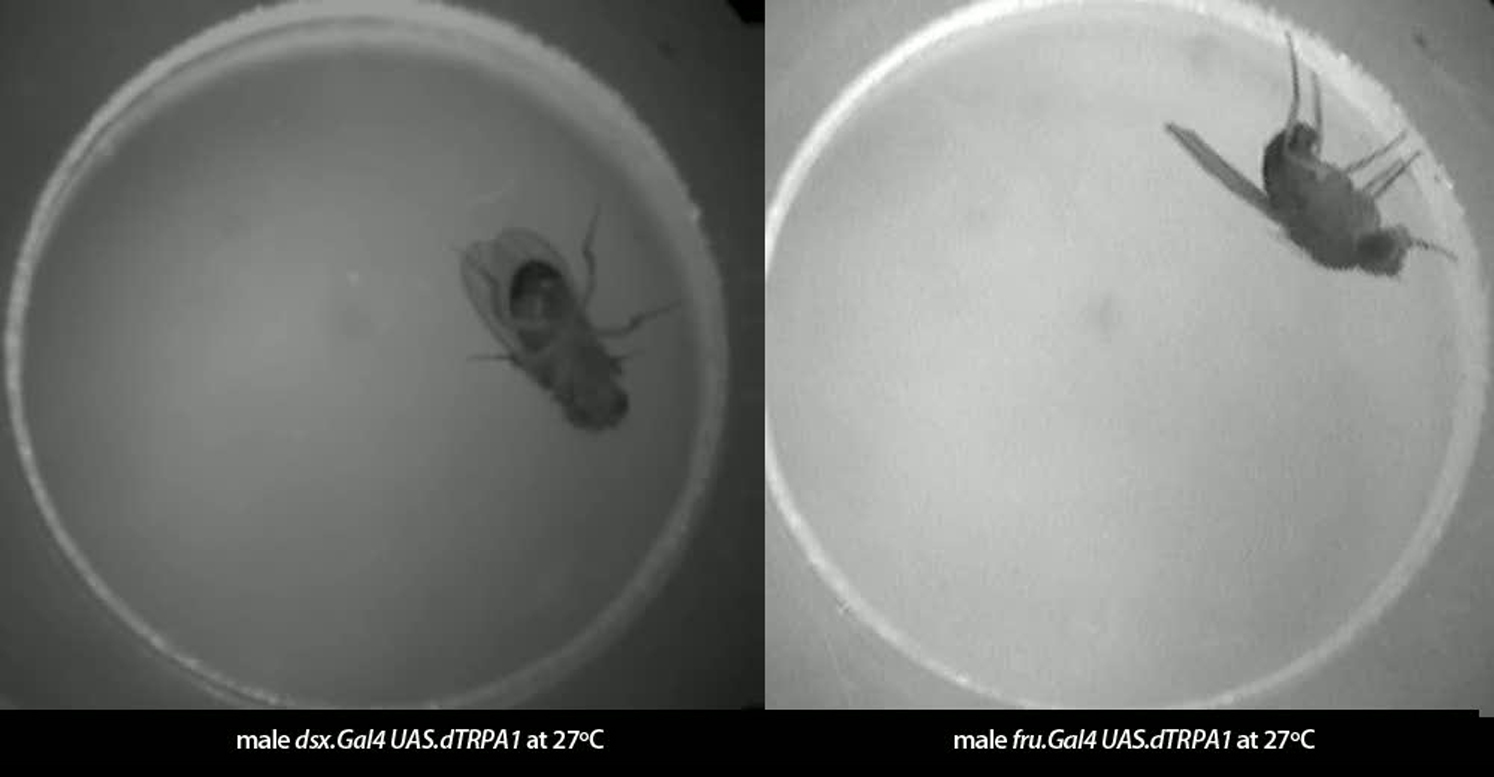

Supplement: Movie S3. Video Clips of a Solitary dsx.Gal4, UAS. dTRPA1 Male, Left, and a Solitary fru.Gal4, UAS.dTRPA1 Male, Right, Related to the Results and Discussion and the Experimental Procedures — At the restrictive temperature, both types of males display several courtship behaviors, including quivering, even though they are solitary. [file mmc3.jpg]

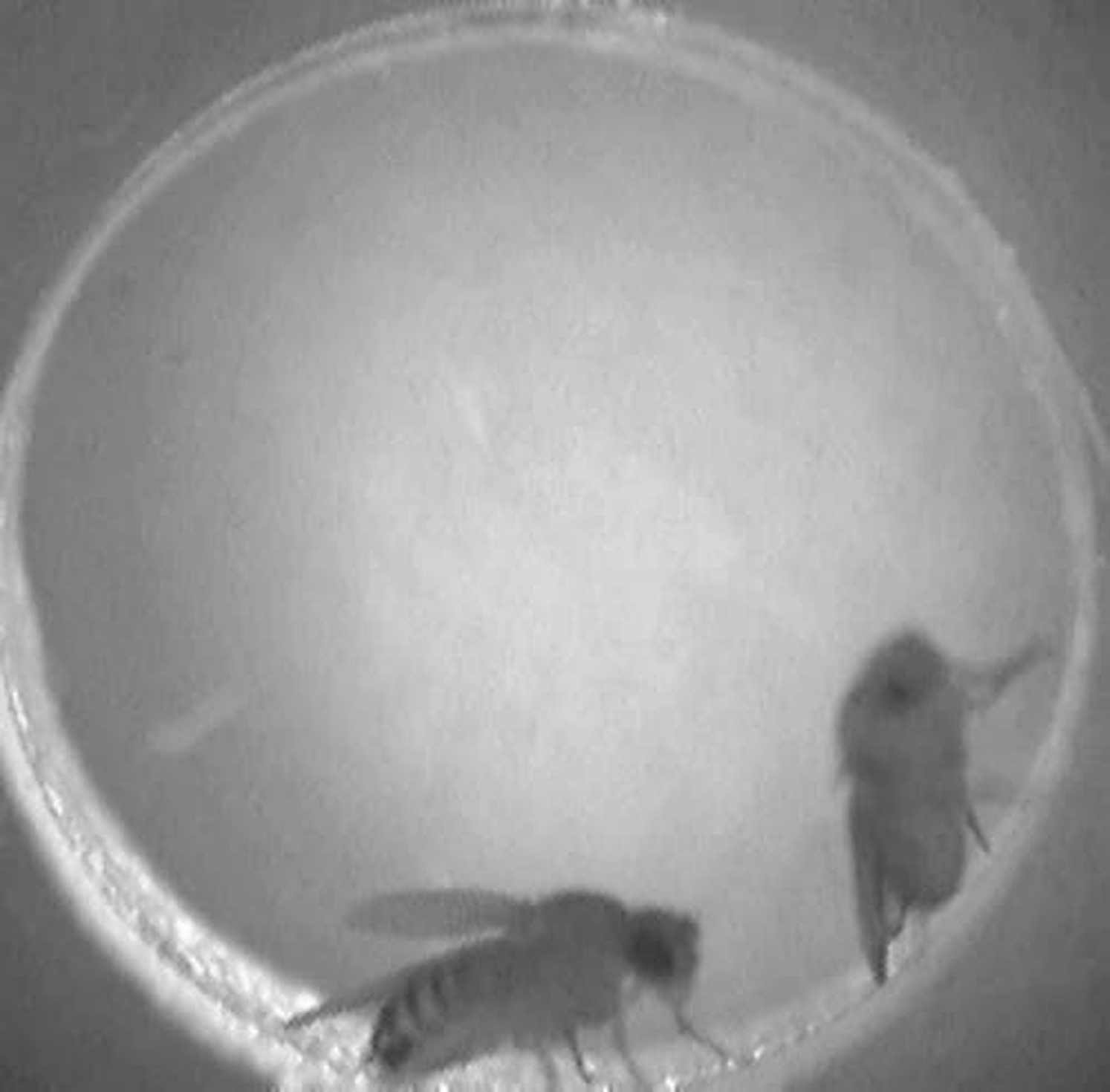

Supplement: Movie S4. Video Clip of Interaction between a Behaviorally Masculinized elav.Gal4, UAS.traIR Female and an Oregon-R Female, Related to Figure 2 — The masculinized female (on the left) courts the wild type female and flutters, and she also displays bouts of quivering. The wild-type female is immobile. [file mmc4.jpg]

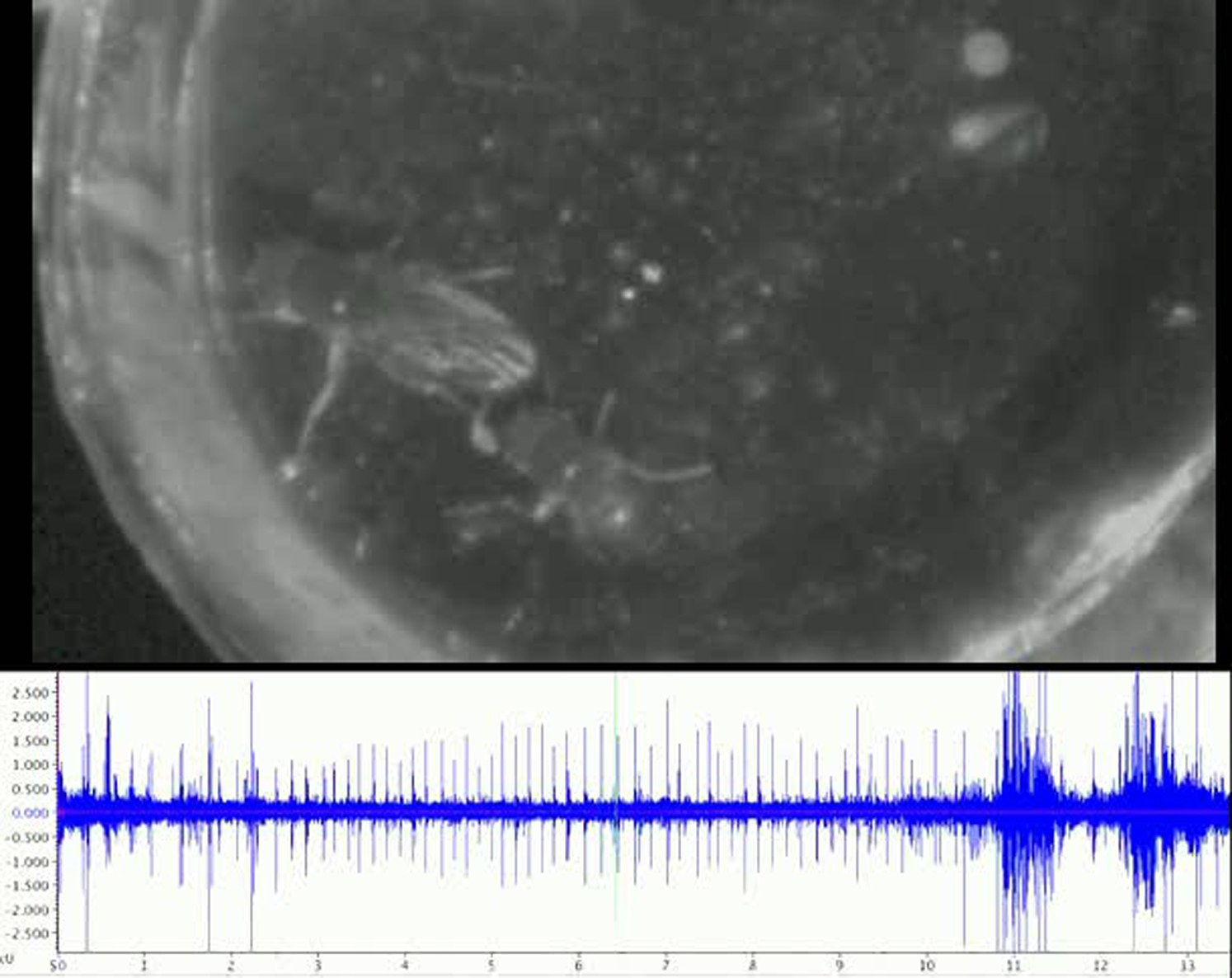

Supplement: Movie S5. Video Clip Showing Simultaneous Quivering and Substrate-Borne Vibrations, Related to Figure 4 — The male quivers and generates characteristic vibrations (see also Figure 3). To reduce interference with the oscillogram, the wings of the male have been amputated. The sound you hear is a transformation of the oscillogram record to audible frequencies; it is only there to help you link the oscillogram record with the video. [file mmc5.jpg]
