# Supplementary material for: Substrate-Borne Vibratory Communication during Courtship in Drosophila melanogaster
Source: Curr Biol. 2012 Nov 20;22(22):2180–5. doi: 10.1016/j.cub.2012.09.042 (PMC3502867; doi:10.1016/j.cub.2012.09.042)
Supplement: Document S1. Figures S1–S4 [file mmc7.pdf]

Current Biology, Volume *22*

Supplemental Information

Substrate-Borne Vibratory Communication during Courtship in *Drosophila melanogaster*

Caroline C.G. Fabre, Berthold Hedwig, Graham Conduit, Peter A. Lawrence, Stephen F. Goodwin, and José Casal

A

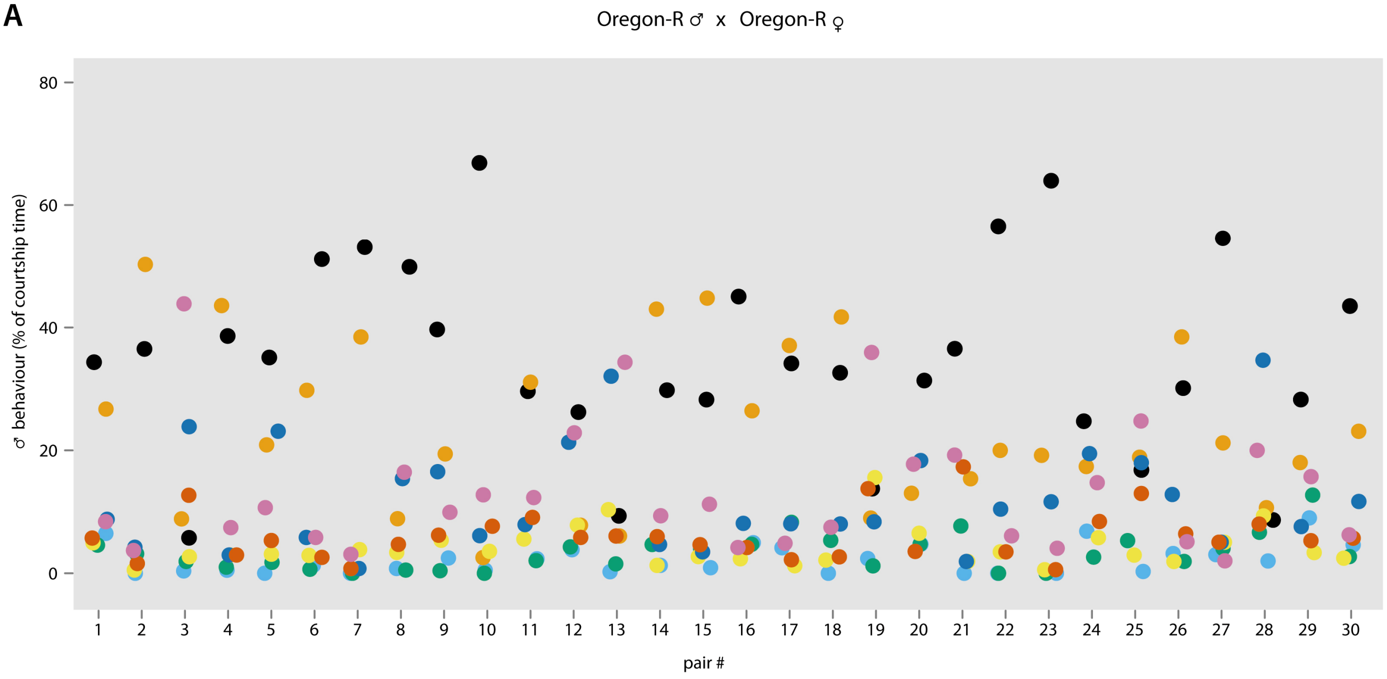

B

		♂ behaviour					
		abdominal quivering	wing fluttering	% of courtship time		bout duration (s)	
				mean	±	mean	±
♀ moving	●	–	–	35.16	5.59	6.50	1.92
	●	+	–	2.21	0.87	1.15	0.27
	●	–	+	23.72	4.77	3.06	0.33
	●	+	+	3.28	1.05	1.39	0.29
♀ immobile	●	–	–	4.19	1.14	2.00	0.33
	●	+	–	12.04	3.08	3.56	0.74
	●	–	+	6.05	1.41	1.81	0.19
	●	+	+	13.35	3.73	2.61	0.37

C

Number of bouts

female mobility (M)	male quivering (Q)	male fluttering (F)	N
moving	–	–	497
moving	+	–	134
moving	–	+	591
moving	+	+	187
immobile	–	–	157
immobile	+	–	264
immobile	–	+	257
immobile	+	+	412

Analysis of chi-square Deviance Table
*Generalised Linear Model (N~M*Q*F)*

	Df	Deviance Resid.	Df	Resid.	Dev. Pr(>Chi)	
NULL	7	619.48				
M	1	40.83	6	578.65	1.659e-10	***
Q	1	102.76	5	475.89	< 2.2e-16	***
F	1	62.70	4	413.19	2.410e-15	***
M:Q	1	401.90	3	11.29	< 2.2e-16	***
M:F	1	9.59	2	1.70	0.001957	**
Q:F	1	0.38	1	1.31	0.535254	
M:Q:F	1	1.31	0	0.00	0.251990	

Signif. codes: 0 '***' 0.001 '**' 0.01 '*' 0.05 '.' 0.1 ' ' 1
Model: poisson, link: log
Response: N
Terms added sequentially (first to last)

D

*Mosaic plots of the Generalised Linear Models (N~M+(or)*Q+(or)*F)*

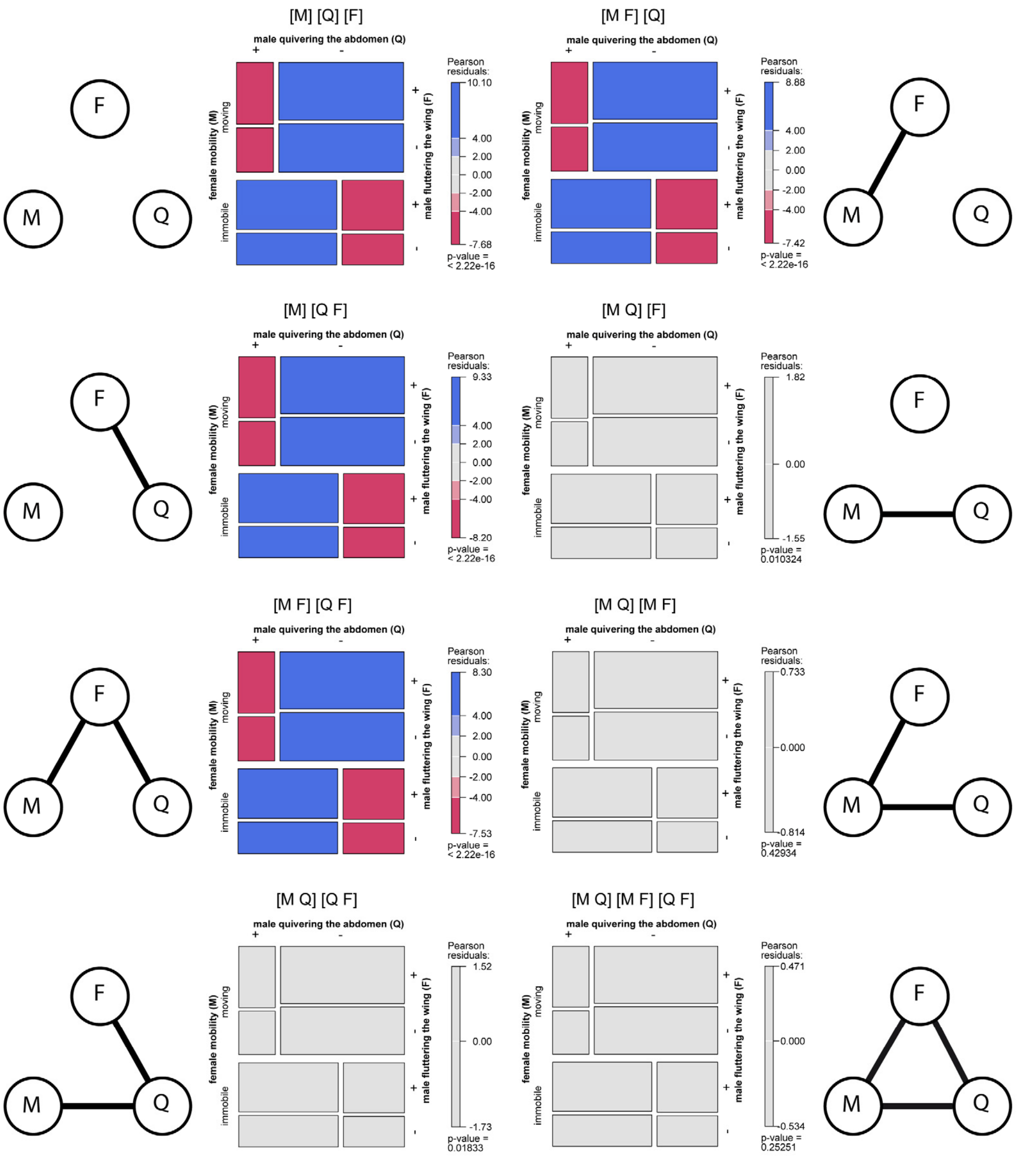

E

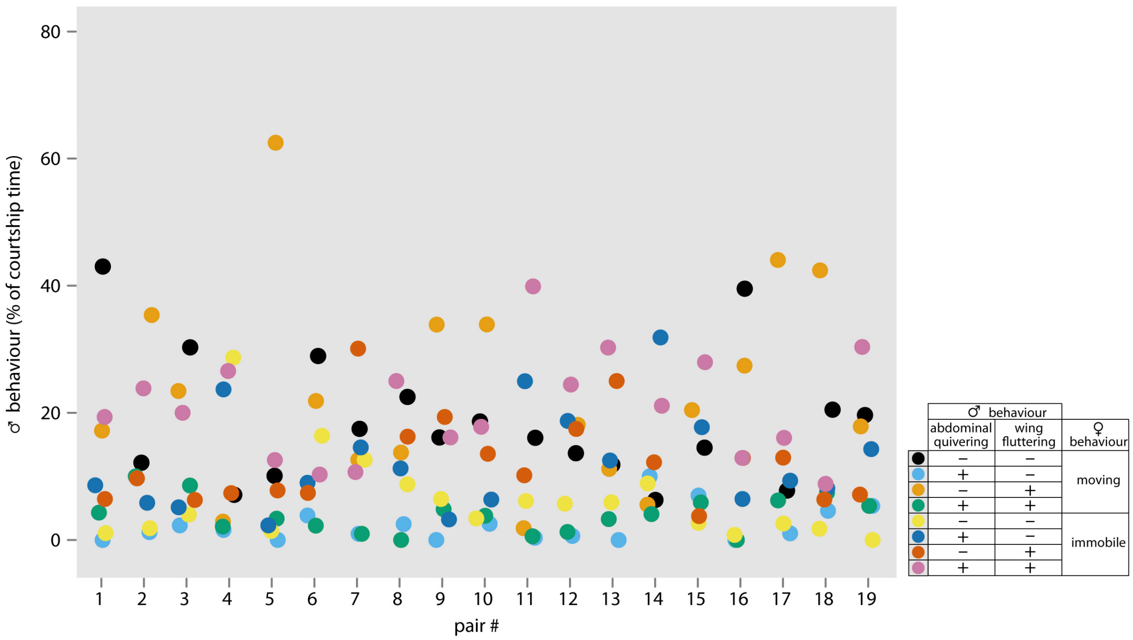

F

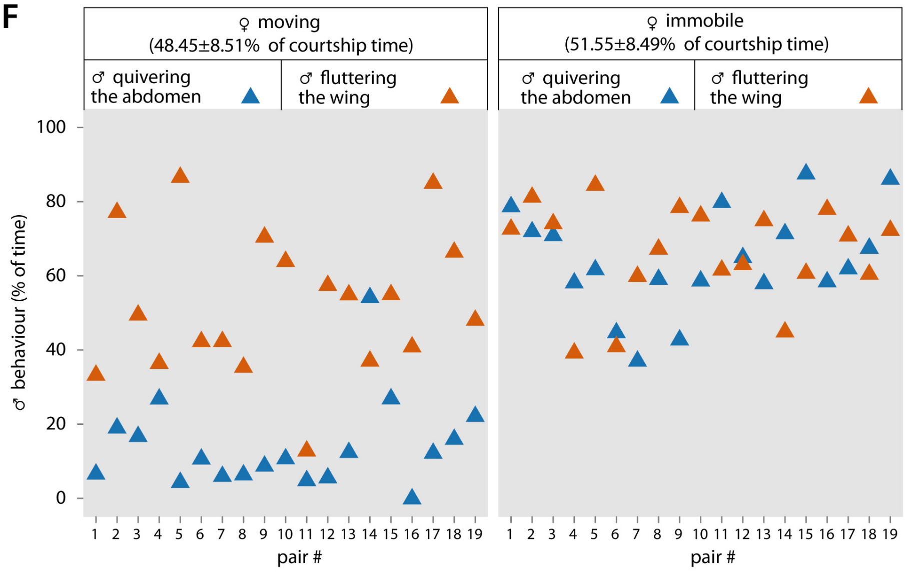

G

	♂ behaviour		
	fluttering the wing only	quivering the abdomen only	fluttering and quivering
♀ moving	44.13 ± 8.31%	5.98 ± 4.04%	8.31 ± 2.39%
♀ immobile	24.76 ± 4.99%	22.55 ± 4.04%	41.71 ± 5.45%

Figure S1. Analysis of courtship in wildtype flies, Related to Figure 1

(**A**) Scatterplot showing the percentage of the total courtship time of selected male and female behaviours from beginning of courtship until copulation for each of 30

pairs (x-axis); percentage of courtship time shown on the y-axis. Colours shown as in (B).

(**B**). Table showing the percentage (grand mean) of the total courtship time corresponding to each of 8 behaviours and the durations (grand mean) of the bouts of each behaviour (± shows interval limits for 95% confidence level). Time resolution is 1 second.

(**C**) The upper contingency table shows a summary of the data selected for statistical analysis of association between different behaviours. Nearly all the bouts lasted 1-10 seconds. A small number of much longer spells of one behaviour (those over twice the average bout length) were noted. Since such behaviour is rare, yet has a gross impact on the mean bout length, these data points were excluded as outliers. N is the number of bouts analysed for the combinations of male and female behaviour shown. The lower table shows the results: for example, for M:Q, a very strong association is found between the male (quivering or not, Q) and female mobility (stopping or not, M). However between the male (fluttering or not, F) and female mobility (stopping or not, M) there was a significant but much weaker association, see M:F. No significant associations were found between the two male behaviours (Q:F) or between all three behaviours (M:Q:F).

(**D**) Mosaic plots of different models of association. The only models that are probable are those that include an association between male quivering behaviour and female mobility behaviour, the most probable model is the conditional dependence [M Q][M F].

(**E**) Ethograms of Canton S: 19 pairs are presented as in Figure S1A.

(**F**) The male behaviour of Canton S pairs presented as in Figure 1A.

(**G**) The table breaks down Canton S male behaviour further as in Figure 1B.

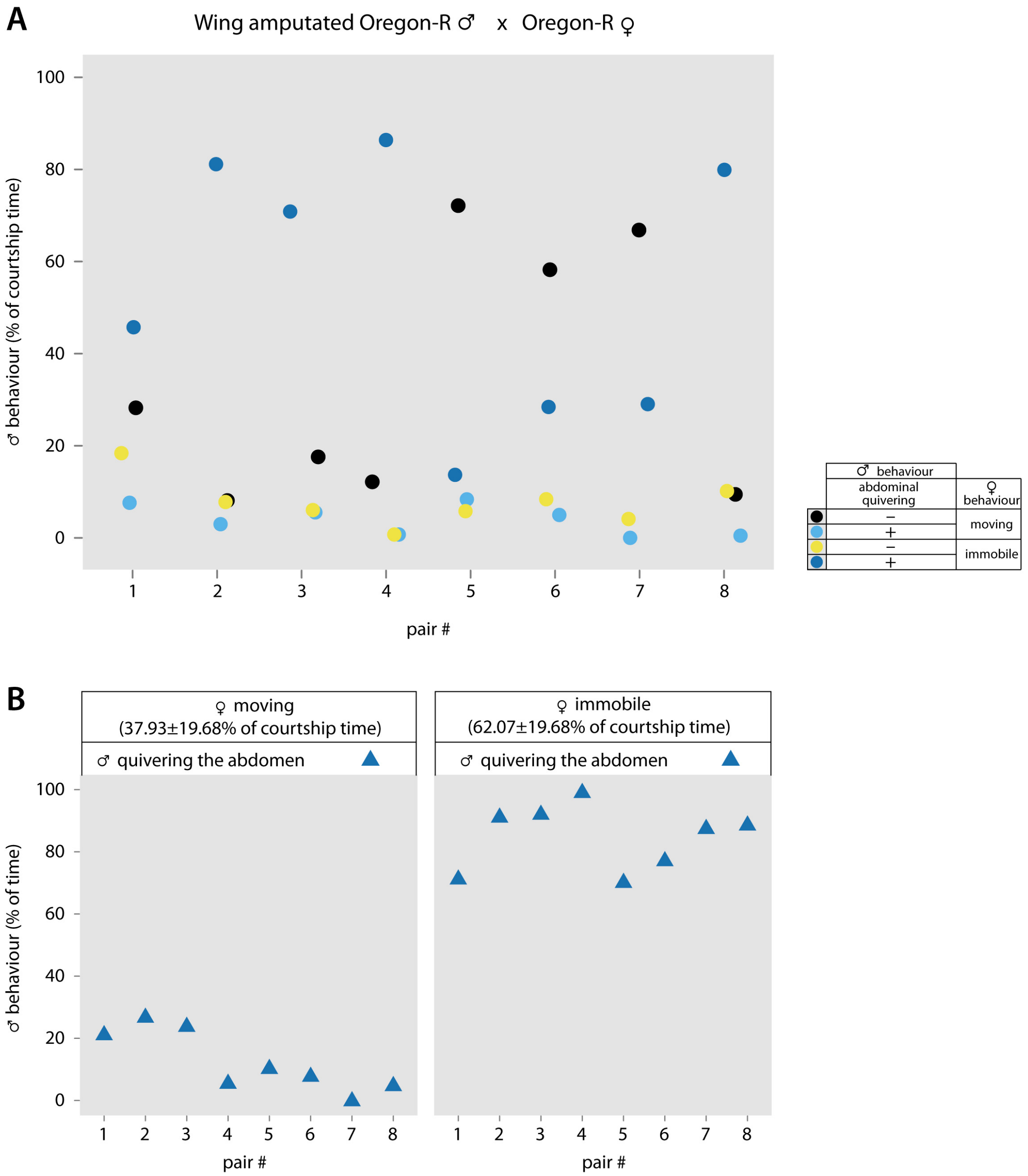

C

	♂ behaviour
	quivering the abdomen only
♀ moving	12.60±6.94%
♀ immobile	84.71±7.29%

D

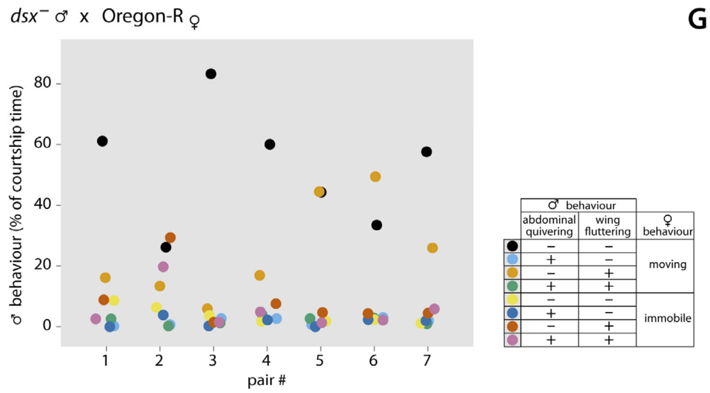

E

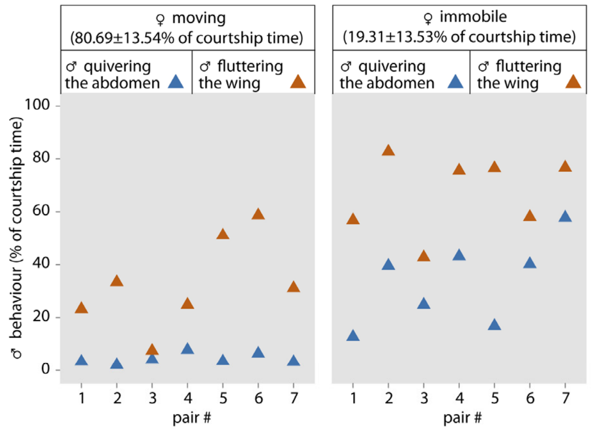

F

	♂ behaviour		
	fluttering the wing only	quivering the abdomen only	fluttering and quivering
♀ moving	30.53±12.66%	2.05±0.90%	2.44±1.11%
♀ immobile	41.83±9.04%	8.42±5.95%	25.31±7.91%

G

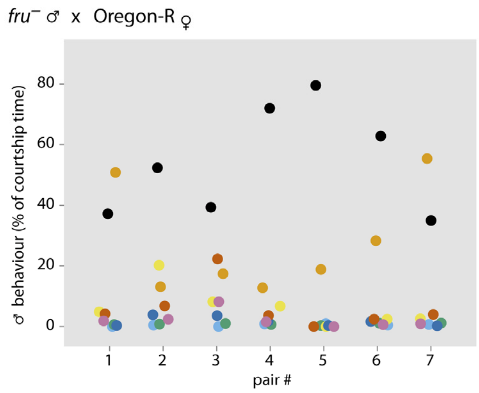

H

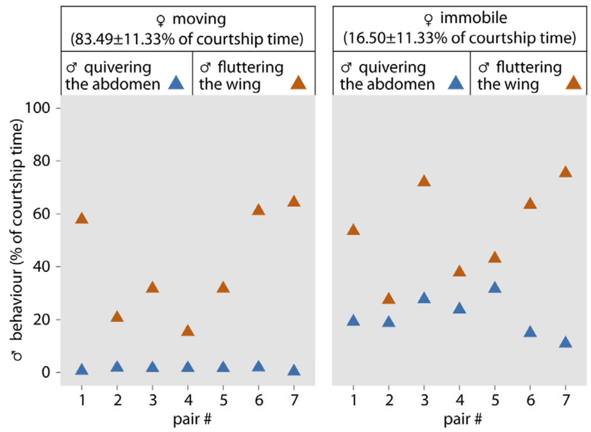

I

	♂ behaviour		
	fluttering the wing only	quivering the abdomen only	fluttering and quivering
♀ moving	36.90±14.05%	0.50±0.31%	0.98±0.32%
♀ immobile	36.12±14.52%	20.44±22.77%	10.60±4.07%

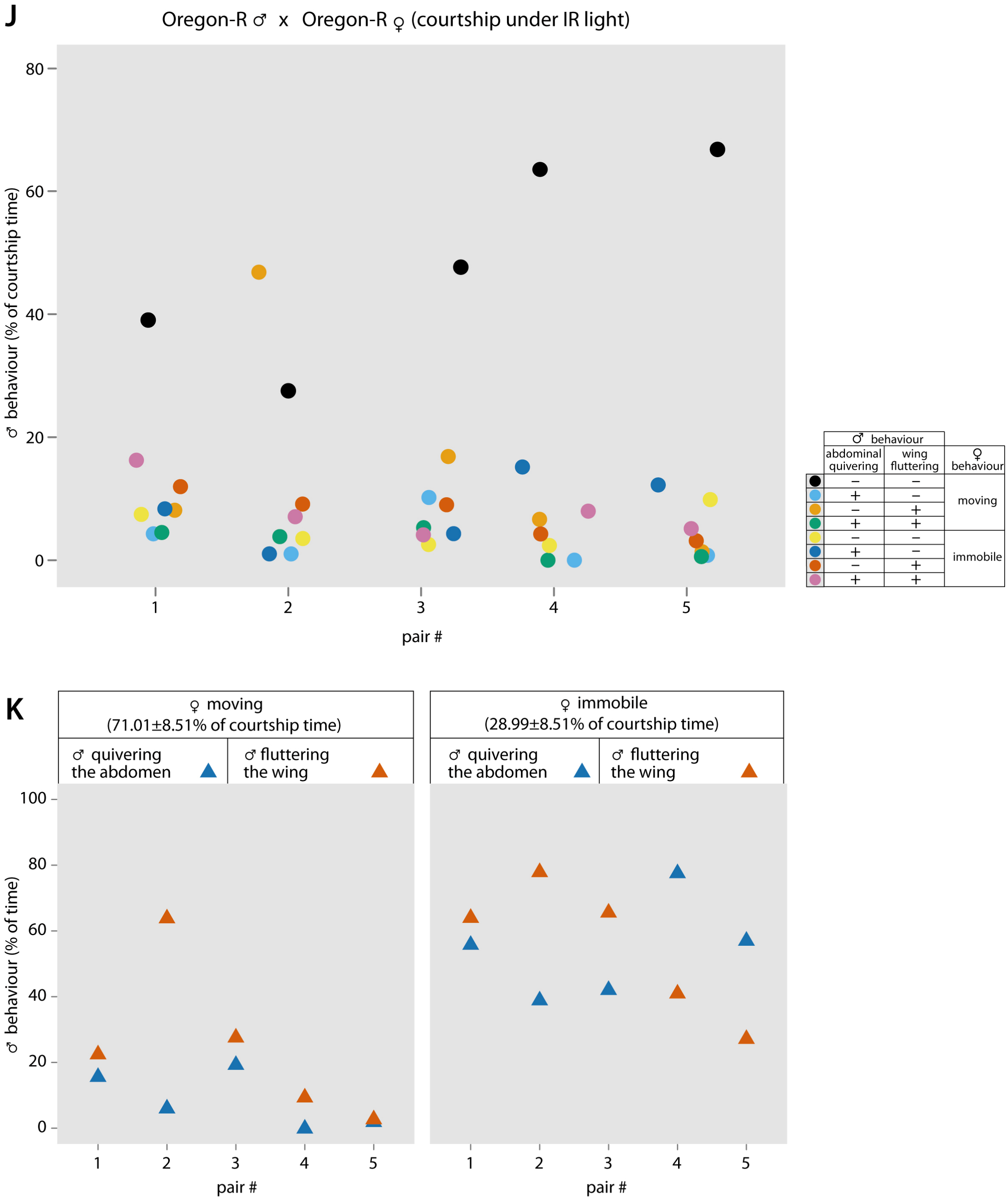

L

	♂ behaviour		
	fluttering the wing only	quivering the abdomen only	fluttering and quivering
♀ moving	21.23 ± 19.54%	4.57 ± 4.80%	4.07 ± 3.09%
♀ immobile	27.33 ± 15.95%	28.19 ± 14.17%	27.04 ± 7.47%

Figure S2. Manipulations that affect the behaviour of males paired with wild type females

(**A-C)** Wings were amputated in Oregon R males: 8 pairs are presented as in Figure S1A-C.
(**D-F)** *dsx*$^{-}$ males: 7 pairs are presented as in Figure S1A-C.
(**G-I)** *fru*$^{-}$ males: 7 pairs are presented as in Figure S1A-C.
(**J-L)** Courtship in the dark by wildtype Oregon R: 5 pairs are presented as in Figure S1A-C.

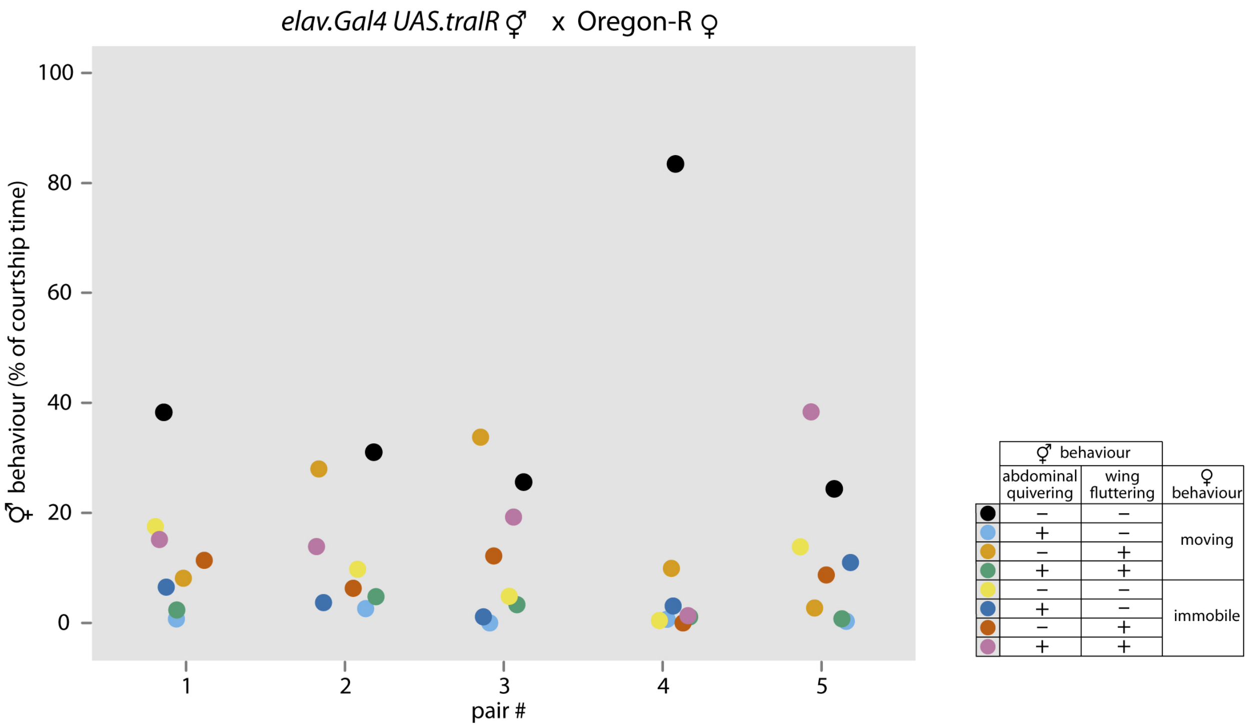

Figure S3. Related to Figure 2

Summary of ethograms of courtship between *elav.Gal4 UAS.traIR* females and wildtype females: 5 pairs are presented as in Figure S1A.

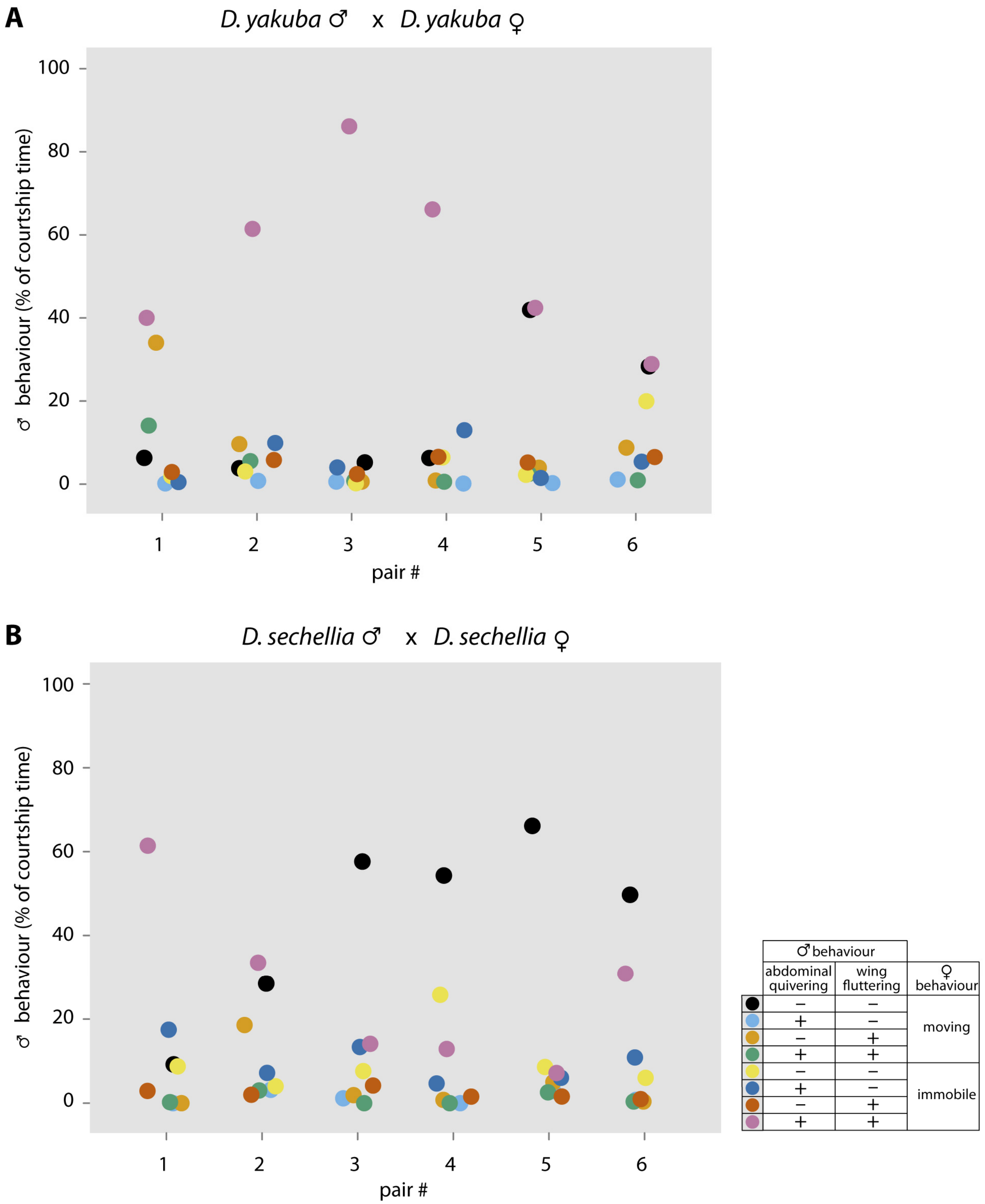

Figure S4. Other species, Related to Figure 4

(**A**) Summary of ethograms of *D. yakuba* courtship: 6 pairs are presented as in Figure S1A.

(**B**) Summary of ethograms of *D. sechellia* courtship: 6 pairs are presented as in Figure S1A.
